# Statistical analysis and significance testing of serial analysis of gene expression data using a Poisson mixture model

**DOI:** 10.1186/1471-2105-8-282

**Published:** 2007-08-02

**Authors:** Scott D Zuyderduyn

**Affiliations:** 1 Victor Ling Laboratory, Department of Cancer Genetics and Developmental Biology, BC Cancer Research Centre, 675 West 10^th ^Ave., Vancouver, Canada; 2 Graduate Program, Department of Biochemistry and Molecular Biology, Faculty of Medicine, University of British Columbia, 2350 Health Sciences Mall, Vancouver, Canada

## Abstract

**Background:**

Serial analysis of gene expression (SAGE) is used to obtain quantitative snapshots of the transcriptome. These profiles are count-based and are assumed to follow a Binomial or Poisson distribution. However, tag counts observed across multiple libraries (for example, one or more groups of biological replicates) have additional variance that cannot be accommodated by this assumption alone. Several models have been proposed to account for this effect, all of which utilize a continuous prior distribution to explain the excess variance. Here, a Poisson mixture model, which assumes excess variability arises from sampling a mixture of distinct components, is proposed and the merits of this model are discussed and evaluated.

**Results:**

The goodness of fit of the Poisson mixture model on 15 sets of biological SAGE replicates is compared to the previously proposed hierarchical gamma-Poisson (negative binomial) model, and a substantial improvement is seen. In further support of the mixture model, there is observed: 1) an increase in the number of mixture components needed to fit the expression of tags representing more than one transcript; and 2) a tendency for components to cluster libraries into the same groups. A confidence score is presented that can identify tags that are differentially expressed between groups of SAGE libraries. Several examples where this test outperforms those previously proposed are highlighted.

**Conclusion:**

The Poisson mixture model performs well as a) a method to represent SAGE data from biological replicates, and b) a basis to assign significance when testing for differential expression between multiple groups of replicates. Code for the R statistical software package is included to assist investigators in applying this model to their own data.

## Background

Serial analysis of gene expression (SAGE) is a technique for obtaining a quantitative, global snapshot of the transcriptome [1]. The method extracts short sequence tags (containing 10, 17, or 22 bp of information, depending on the protocol) from each messenger RNA; these are serially ligated, cloned and sequenced, and can then be counted to obtain a profile [1-3]. SAGE has been used to study the transcriptome of a variety of tissue and cell types from a diverse set of organisms. The technique was originally conceived to study the cancer transcriptome, and has been utilized extensively to do so.

As a counting technology, SAGE produces profiles consisting of a digital output that is quantitative in nature. For example, a statement can be made with reasonable certainty that a SAGE tag observed 30 times in a library of 100,000 tags corresponds to a transcript that comprises 0.03% of the total transcriptome; the same statement cannot be made reliably with analog values, like that obtained from a microarray. Accordingly, a reliable statistical model should account for the discrete, count-based nature of SAGE observations. When testing for differential expression between groups, where each group can contain multiple libraries, statistical methods that incorporate a continuous probability distribution (e.g. the Normal distribution assumed by Student's *t*-test) should be avoided. Indeed, such tests require tag counts be normalized by division with the total library size; this removal of library size from the set of sufficient statistics discards an informative facet of the data.

The sampling of SAGE tags can be modeled by the Binomial distribution which describes the probability of observing a number of successes in a series of Bernoulli trials. Here, the library size corresponds to the number of trials and the count of a particular tag is the number of successful trial outcomes. When the probability of an event is small, the Binomal distribution approaches the Poisson distribution as the number of trials increases. This is the case for SAGE (since the tag counts are small relative to a large library size), so the form of the Poisson and Binomial distribution is essentially the same. A fortunate characteristic of both of these distributions is that they are a function of a single parameter only, since the variance in observed data is directly calculable from the mean.

However, in practice, the variance of SAGE data is often larger than can be explained by sampling alone. Several authors have attributed this effect, termed "overdispersion", to a latent biological variability [4-6]. [4] refers to this as "between"-library variability, as opposed to "within"-library variability caused by sampling. Examples of factors that could contribute to this variability are numerous, including: sample preparation or quality, artefacts intrinsic to the library construction protocol, differences in gene transcription due to environment, or the intrinsic stability or regulatory complexity of transcription at a particular locus. This will adversely affect statistical analysis because additional variance results in an overstated significance. Procedures for using hierarchical models which incorporate a continuous prior distribution to explain the excess variance have been presented for both the Binomial (*viz*. beta-binomial using logistic regression [5], t_w_-test [4], or Bayes error rate [7]) and Poisson (*viz*. negative binomial a.k.a. hierarchical gamma-Poisson using log-linear regression [6]) distributions. Attempts to use the log-normal and inverse-Gaussian as prior distributions (both of these have longer tails) did not show an appreciable improvement and are computationally difficult to fit (data not shown).

**Figure 1:**
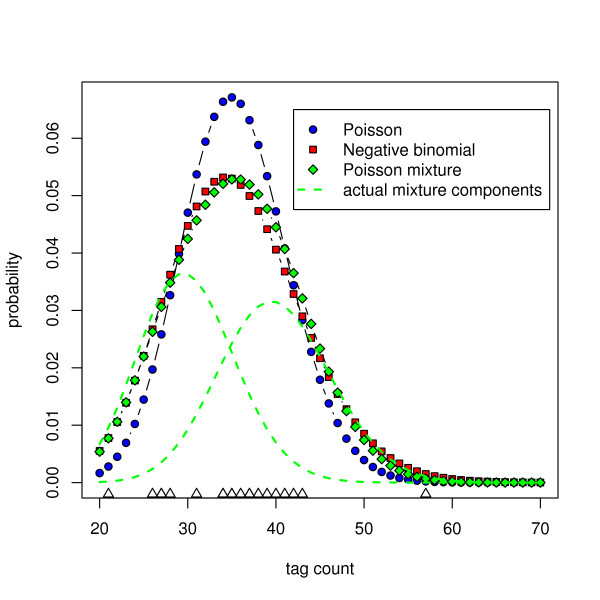
**Probability density of several models applied to data generated from two Poisson components**. 10 observations were randomly drawn from each of two Poisson distributions, one with a mean of 30, the other 40. The values drawn from the first component were (40, 34, 37, 28, 31, 21, 41, 27, 34, 27) and the values drawn from the second component were (36, 42, 26, 57, 43, 37, 38, 39, 35, 35). The probability densities are shown for a single Poisson distribution, the negative binomial distribution, and a two-component Poisson mixture distribution using maximum likelihood estimates (see Methods). The probability densities of the individual Poisson components from which the data were actually drawn are also shown. The individual observations are represented by triangles at the bottom of the plot.

Here, it is argued that the excess variation is due to a mixing of two or more distinct Poisson (or Binomial) components, and this mixing is the predominant source of total variation. This assumption corresponds to a finite mixture model, which have found wide applicability in several fields (for a general introduction, McLachlan and Peel is a good source [8]). To illustrate, consider a tag from ten SAGE libraries of equal size (e.g. 100,000 tags) that has observed counts where half are realizations of an expression of 0.0003 and the other half of 0.0004. As a result, the probability distribution of observing a particular tag count will be a combination of these two components (Figure 1). Note the similarity between the shapes of the probability distributions estimated from a fitted negative binomial (which assumes sampling variability drawn from a latent biological variability) and a Poisson mixture model (which assumes a set of independent components, each having sampling variability only).

If the Poisson mixture model is an accurate foundation to explain SAGE observations, it is attractive for several reasons. First, this approach does not rely on a vague and continuous prior distribution to explain additional variance. Rather, the model asserts that a gene's expression level will take on one of a number of distinct states. Second, overdispersed models applied to SAGE data tend to show a wide range of excess variation; in many cases, far greater than can be attributed to counting. This is a troubling prospect for studies that utilize a limited number of libraries (e.g. pair-wise comparisons), since the observed count may differ wildly from the underlying expression. If a mixture model provides an improved fit to SAGE data, this concern would be assuaged. Finally, mixture models, by nature, allow for the concept of subsets (or latent classes) in the expression values of each tag. Dysregulation of genes in disease processes such as cancer are often observed in only a proportion of profiled samples, and these will be naturally identified during model fitting. This property can also be utilized to identify sets of co-expressed genes.

## Results

### Goodness of fit

In order to evaluate the efficacy of a mixture model approach, a comparison of the goodness of fit of this and previously described models on 15 sets of biological replicates from publicly available SAGE data was performed (see Methods).

Goodness of fit was assessed for: 1) the canonical log-linear (Poisson) model, 2) negative binomial (i.e. hierarchical gamma-Poisson or overdispersed log-linear) model, and 3) k-component Poisson mixture model (see Methods for a brief description of each). Since maximum likelihood estimation (MLE) is used to fit each of these models, the log-likelihood was the basis for assessing relative goodness of fit. A comparison of the Akaike information criterion (AIC) [9] and Bayesian information criterion (BIC) [10] (both of which use the log-likelihood and a term to penalize a model for estimating a larger number of parameters) was performed on each of the datasets (Table 1).

**Table 1:** Comparison of model fits to a single group of biological replicates

				**mean AIC**			**mean BIC**		
	**N**	**tags**	**k**	**Poisson**	**Negbin**	**Mixture**	**Poisson**	**Negbin**	**Mixture**

**BRAIN**									
astrocytoma	14	1141	2.6	238.2	105.2	103.6	238.9	106.5	106.3
ependymoma	10	1205	2.3	152.4	80.9	75.0	152.7	81.5	76.1
glioblastoma	7	1197	2.3	139.5	57.6	53.0	139.4	57.5	52.8
medulloblastoma	18	1045	2.7	280.6	128.7	128.7	281.5	130.5	132.6
normal	8	1099	2.4	156.8	68.0	59.8	156.8	68.2	60.1
**AML**									
inv(16)	5	900	1.7	68.9	39.3	37.6	68.5	38.5	36.7
t(8;21)	5	1037	1.3	52.3	34.1	33.5	51.9	33.3	32.9
t(15;17)	5	709	1.8	127.7	46.0	38.9	127.3	45.2	37.9
t(9;11) *de novo*	4	954	1.8	58.5	34.6	30.1	57.9	33.4	28.5
t(9;11) treatment	3	1061	1.5	42.9	32.0	20.9	42.0	30.2	19.1
**BREAST**									
normal	6	1259	1.8	71.6	43.9	41.6	71.4	43.5	41.0
DCIS	4	598	1.3	25.5	24.0	21.0	24.9	22.8	20.1
invasive	3	1069	2.0	60.4	27.8	22.8	59.5	26.0	20.1
**SKIN**									
normal	4	1015	1.6	33.8	24.6	22.2	33.2	23.4	20.9
melanoma	3	992	1.8	38.2	24.0	19.6	37.3	22.2	17.4

SAGE tag counts from fifteen sets of biological replicates were fit to log-linear (Poisson), negative binomial (overdispersed log-linear), and Poisson mixture models. The table contains the number of replicates (N), tags tested, and mean number of mixture components (k). For each model, mean goodness of fit scores calculated using Akaike's Information Criterion (AIC) and Bayesian Information Criterion (BIC) are shown. For both scores, a lower value indicates a better fit.

As expected, the canonical Poisson model, which does not account for excess variance, performs poorly in all cases. The Poisson mixture model consistently outperforms the negative binomial model regardless of the metric used. The competitiveness of the negative binomial model is perhaps not surprising since a comparison of the fit of these two models to simulated data indicates that the negative binomial can often fit better to data generated from a two-component Poisson mixture. This becomes more problematic as the component means draw closer (data not shown, Figure 1 is a good example). However, several hypotheses can be tested to further strengthen the case for the mixture model approach. These are considered in turn.

### Tags with ambiguous mappings are represented by a greater number of components

Consider an idealized situation where a gene's expression can take on one of two states (and can therefore be modelled by a two-component Poisson mixture). A significant proportion of SAGE tags are ambiguous (correspond to more than one gene) and, under the idealized model, would result in tag counts that are modelled by 2^g ^components (where g is the number of expressed genes the tag corresponds to). Therefore, the number of components in the mixture should be higher for ambiguous tags.

Simply partitioning the data into ambiguous and unambiguous tags and comparing the number of components is unlikely to be informative since, for any given ambiguous tag, it is not known how many of the possible genes are actually expressed. However, two normal brain libraries used in this study were generated using LongSAGE (GSM31931 and GSM31935), which provides 17 base pairs of information rather than 10. The tag sequences in these libraries were shortened before inclusion in the normal brain dataset used in the previous section. However, by comparing the shortened tag list to the original library, tags that actually correspond to two or more LongSAGE tag sequences (and presumably represent different transcripts) were identified. Tags counts of one or two were considered artefacts of PCR amplification or sequencing and were not used in this determination.

The number of ambiguous and unambiguous tags was tallied for each estimated number of components (Table 2). Ambiguous tags are represented more highly in the set of model fits that consist of a larger number of components. This effect, which is statistically significant, is consistent with the mixture model hypothesis.

**Table 2:** Mean number of mixture model components

**Library**	***k***	**Unambiguous**	**Ambiguous**	**Significance**
GSM31931	1	93	0	*p *< 2.2E-16 (*χ*^2 ^= 134.1; df = 4)
	2	405	15	
	3	210	32	
	4	27	12	
	5	5	12	
GSM31935	1	74	34	*p *= 1.8E-6 (*χ*^2 ^= 32.1; df = 4)
	2	317	246	
	3	149	171	
	4	17	30	
	5	3	14	

Expressed ambiguous and unambiguous 10 base pair tags for two LongSAGE libraries were distinguished based on the number of 17 base pair sequences that give rise to the same tag. The tags in each of these two groups were binned according to the number of estimated mixture components. The Chi-square statistic was used to test the null hypothesis that these two groups are equivalent.

### Component assignment of libraries is non-random

If the mixture model approach holds, then the Poisson components should cluster the libraries into recurring groups. Such an enrichment of certain component assignments would be expected for a number of reasons. Two possibilities are: a) if one or more libraries are mislabelled, the tag expression in those libraries should show a preferential assignment to a separate component; and b) if the genes corresponding to a set of tags are co-expressed, the component assignment should be similar amongst these genes. Conversely, if the negative binomial model is more appropriate then component assignments should essentially be random, since the distribution assumed to give rise to biological variability is continuous and unconditional.

For each of the datasets, the component assignments for tags where the estimated number of components is two were tallied. The individual assignment was based on the component with the highest posterior probability, given a tag count and library size. In all cases, there were a significant number of tags where the parent libraries were partitioned into the same two components (Table 3). For example, in the AML libraries containing the cytogenetic abnormality t(8;21), of the 225 tags that had expression that could be fit to two Poisson components, 110 were partitioned in the form -++-- (*p *= 4.5E-67; Binomial test). In other words, almost half of the tags that fit to two components were assigned to a single component configuration (for 5 libraries, (2^5^/2)-1 = 15 such configurations are possible).

**Table 3:** Top component memberships

**Dataset**		
**Component assignment**	**Freq.**	***p*-value**

**BRAIN**		
astrocytoma (N = 14 n_k = 2 _= 454)		
-+------------	14	4.3E-28
---+++++++++++	12	2.7E-23
ependymoma (N = 10 n_k = 2 _= 544)		
-+--------	16	6.0E-14
--+-------	15	9.3E-13
glioblastoma (N = 7 n_k = 2 _= 607)		
---+---	48	3.2E-19
-----+-	42	5.2E-15
medulloblastoma (N = 18 n_k = 2 _= 438)		
-----------------+	4	1.5E-9
-----------+-----+	4	1.5E-9
normal (N = 9 n_k = 2 _= 588)		
-----+--	41	7.4E-37
-----+-+	21	7.6E-14
**AML**		
inv(16) (N = 5 n_k = 2 _= 387)		
-++++	110	2.5E-39
-+-++	36	0.028
t(8;21) (N = 5 n_k = 2 _= 387)		
-++--	110	2.5E-39
-++-+	36	0.028
t(15;17) (N = 5 n_k = 2 _= 225)		
-++--	110	4.5E-67
-++-+	36	1.0E-6
t(9;11) *de novo *(N = 4 n_k = 2 _= 502)		
-+-+	143	1.6E-16
-+--	132	1.4E-12
t(9;11) treated (N = 3 n_k = 2 _= 405)		
-++	216	1.1E-16
**BREAST**		
normal (N = 6 n_k = 2 _= 571)		
---+--	82	3.6E-29
-----+	65	4.0E-18
DCIS (N = 4 n_k = 2 _= 154)		
--++	97	1.4E-43
-+++	41	4.5E-5
invasive (N = 3 n_k = 2 _= 765)		
-+-	337	4.6E-10
**SKIN**		
normal (N = 4 n_k = 2 _= 500)		
---+	215	1.8E-54
melanoma (N = 3 n_k = 2 _= 650)		
-+-	405	2.1E-51

For each set of biological replicates, the top one or two component states were selected from tags where the estimated number of components is 2. One component was represented with -, the other with + (i.e. -+- is equivalent to +-+). The significance was calculated using a zero-truncated Binomial test. The number of possible ways for the libraries to be assigned to the two components is (2^N^/2)-1, where N is the number of libraries.

### Determining differentially expressed genes

In previously described overdispersed models, the identity of a library is included *a priori *as a model covariate. Significance is then determined by testing the null hypothesis that the fitted *β *coefficient for this covariate is zero [5,6]. A Bayesian significance score has also described, although this was developed using a beta-binomial model [7]. In contrast, the Poisson mixture model does not require the identity of the libraries be included (although the addition of such covariates is possible). Rather, once a mixture model has been fit, the posterior probabilities of membership in a particular component given the observed tag counts can be used to determine how well the components can differentiate between two or more sample types (e.g. normal versus cancer). Here, a score is presented based on the confidence that a sample is of type *ω *given that it arises from component(s) k. Using Bayes Theorem, one can derive the following expression [see Additional file 1]

P(ω|k)=∑jωτjk/∑jτjk
 MathType@MTEF@5@5@+=feaafiart1ev1aaatCvAUfKttLearuWrP9MDH5MBPbIqV92AaeXatLxBI9gBaebbnrfifHhDYfgasaacH8akY=wiFfYdH8Gipec8Eeeu0xXdbba9frFj0=OqFfea0dXdd9vqai=hGuQ8kuc9pgc9s8qqaq=dirpe0xb9q8qiLsFr0=vr0=vr0dc8meaabaqaciaacaGaaeqabaqabeGadaaakeaacqWGqbaucqGGOaakiiGacqWFjpWDcqGG8baFcqWGRbWAcqGGPaqkcqGH9aqpdaWcgaqaamaaqahabaGae8hXdq3aaSbaaSqaaiabdQgaQjabdUgaRbqabaaabaGaemOAaOgabaGae8xYdChaniabggHiLdaakeaadaaeqbqaaiab=r8a0naaBaaaleaacqWGQbGAcqWGRbWAaeqaaaqaaiabdQgaQbqab0GaeyyeIuoaaaaaaa@4781@

where *ω *is the set of libraries corresponding to some label of interest (e.g. normal or cancer) and *τ*_jk _is the posterior probability of the tag count from library j arising from component(s) k. Using this expression, one can determine which tags have a set of mixture components that are closely linked with the sample type(s) of interest.

**Figure 2:**
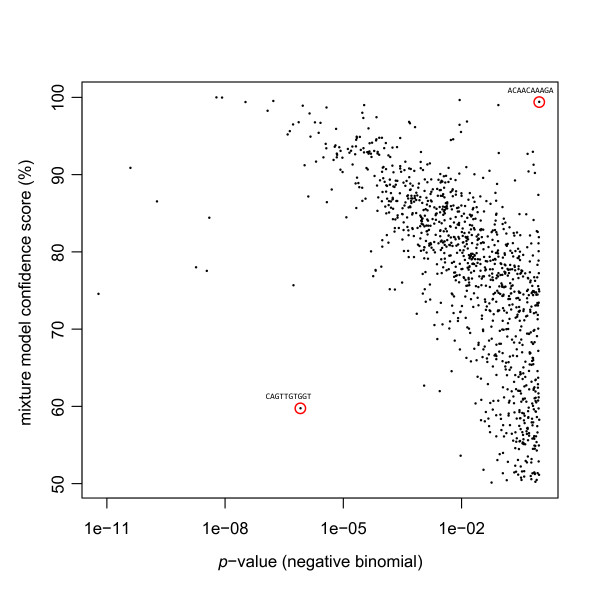
**Comparison to significance scores for a test of differential expression calculated using a negative binomial model**. Using the tag counts from 8 normal brain libraries versus 10 ependymoma libraries, differential expression between these two sample types was assessed using two methods. Plotted are the significance scores calculated for a negative binomial model versus a Poisson mixture model. The negative binomial (x-axis) is a *p*-value, so smaller values are more significant. The Poisson mixture (y-axis) is a confidence score, so larger values are more significant. Circled are two examples of SAGE tags where one model shows significance while the other does not.

To illustrate, SAGE libraries from normal brain (n = 8) and ependymoma (n = 10) (a type of brain tumour) were analyzed using both the overdispersed log-linear and Poisson mixture models. In the former case, significance was calculated using the method described in [5] (see also example R code in Methods). In the latter case, the method described above was used. A plot of the two sets of scores shows a moderate correlation and tags that are found highly significant in one test tend to be so in the other (Figure 2).

**Figure 3:**
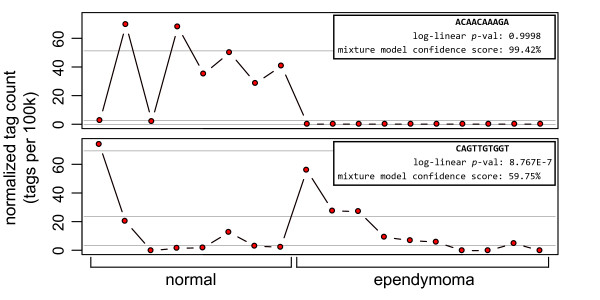
**Counts for two tags assessed using a negative binomial model and the Poisson mixture model where one model shows significance and the other does not**. The figure is divided to show separate plots of the expression level of two tags observed in 8 normal brain libraries and 10 ependymoma libraries. The x-axis is the normalized expression (count/library size*100,000) and the y-axis is divided into the two sample types. In the top plot, the negative binomial model is not significant and the Poisson mixture is significant; in the bottom plot, the situation is reversed. Light gray guide lines denote the expected expression level of the Poisson components.

However, a number of observations are found significant using the overdispersed log-linear model and not the Poisson mixture model, and vice versa. A closer look at the most extreme examples illustrates the superior performance of the mixture approach (Figure 3). In the first example, tag ACAACAAAGA seems clearly expressed in normal libraries, but is completely abolished in the ependymoma libraries. However, according to the overdispersed model, the observation is not at all significant (*p *= 0.9998). The mixture model, however, produces a confidence score of 99.42%, which suggests this tag is highly informative with respect to sample type. This example demonstrates the difficulty that the log-linear model has with fitting groups where tag counts are zero, a problem that is even more pronounced when using a logistic regression model (for a more thorough discussion of this problem see [6]).

In the second example, tag CAGTTGTGGT clearly has increased expression in some libraries from both the normal and ependymoma groups. However, according to the overdispersed model, the observation is highly significant (*p *= 8.8E-7). The mixture model, however, produces a confidence score of 59.8% which is only nominally better than chance. This example demonstrates how the log-linear model seems to downweight the occasional extreme observation in one group, even if it is in agreement with observations in the other group. This can result in candidate lists based on the log-linear significance containing tags that have extreme observations that occur at a higher rate in one group over another, which are typically of little interest.

**Figure 4:**
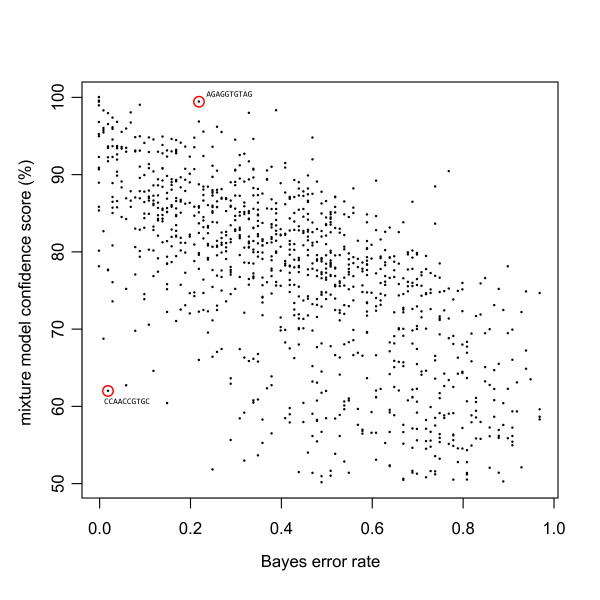
**Comparison to Bayes error rate for a test of differential expression calculated using a beta binomial model**. Using the tag counts from 8 normal brain libraries versus 10 ependymoma libraries, differential expression between these two sample types was assessed using two methods. Plotted are the Bayes error rate described in [7] versus a Poisson mixture model confidence score. For the Bayes error rate (x-axis) smaller values are more significant. The Poisson mixture (y-axis) is a confidence score, so larger values are more significant. Circled are two examples of SAGE tags where one model shows significance while the other does not.

**Figure 5:**
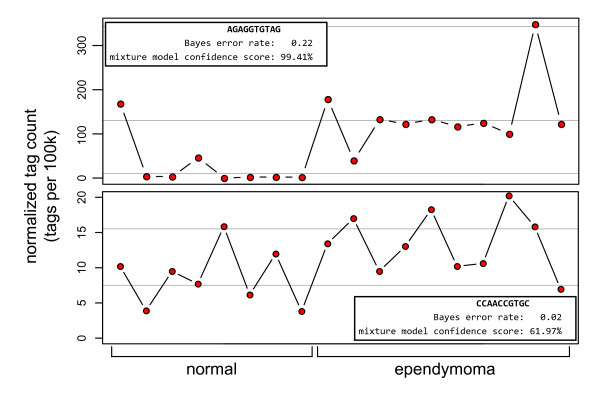
**Counts for two tags assessed using a Bayes error rate and the Poisson mixture model where one model shows significance and the other does not**. The figure is divided to show separate plots of the expression level of two tags observed in 8 normal brain libraries and 10 ependymoma libraries. The x-axis is the normalized expression (count/library size*100,000) and the y-axis is divided into the two sample types. In the top plot, the Bayes error rate is not significant and the Poisson mixture is significant; in the bottom plot, the situation is reversed. Light gray guide lines denote the expected expression level of the Poisson components.

Similar results were obtained when comparing to the Bayes error rate described in [7]. Again, a moderate correlation is seen and tags found highly significant in one test tend to be so in the other (Figure 4). Overall, the Bayes error rate is in better agreement with the mixture model confidence score and appears to be more robust in assessing tags with zero counts in one group. However, the assumption of a hierarchical model (in this case, a beta-binomial) used to calculate the Bayes error rate versus a Poisson mixture model results in differences between the two methods. Two examples, analogous to those described above, are highlighted (Figure 5). In both cases, the Poisson mixture model appears to give confidence values that are in better agreement with the observations.

## Discussion

The exploration of statistical approaches to SAGE analysis is important since the number of studies using the technology has resulted in a continuing rise in the amount of available data. The notion of sampling variability being the predominant source of "within"-library variability and distinct components being the predominant source of "between"-library variability is reassuring for investigators who choose the SAGE technique to obtain a comprehensive profile of gene expression in a limited number of samples. Nevertheless, there is certainly a contribution by a latent biological variability as evidenced by the increased performance of the negative binomial as the number of libraries increases. However, this work demonstrates that a simple overdispersed model may overstate this effect, and that certainly there is a clustering of expression into distinct components, which are then sampled. This is consistent with the view of gene transcription for any one locus consisting of (possibly several) inactivated or activated state(s). The same idea holds for some known mechanisms of genetic disease, such as loss of heterozygosity (LOH) or amplification of a particular locus (e.g. cancer).

For this reason, it is recommended that investigators try the mixture model approach in comparisons of groups of biological replicates. Failing this, some of the difficulties that can be encountered with the negative binomial model can be lessened by: a) setting a tolerance for how much overdispersion (*ϕ*) is acceptable in a final list of candidate tags, although such a cutoff would be somewhat arbritrary; and b) add a small value to the tag count to avoid the problems the model has with groups consisting of many zero counts. One strategy is to assume equal odds that the next tag drawn is the one of interest by adding 1 to the count, and 2 to the library size (i.e. (count+1)/(size+2)) (K. Baggerly, *personal communication*).

In the future, it may be worthwhile to combine both approaches by defining a negative binomial mixture model. However, at this point, such an approach is unlikely to provide significant improvement given the small number of libraries in a typical set of available biological replicates. In addition, applying the concept of "information sharing" between tags may provide estimates of statistically informative variables that apply library-wide, and could be utilized to improve the power of the method described in this paper [11,12].

## Conclusion

The Poisson mixture model appears to be a rational means to represent SAGE data that are biological replicates and as a basis to assign significance when comparing multiple groups of such replicates. The use of a mixture model can improve the process of selecting differentially expressed genes, and provide a foundation for *ab initio *identification of co-expressed genes and/or biologically-relevant sample subsets.

## Methods

### Test datasets

Test datasets were obtained from the Gene Expression Omnibus (GEO) [13] and reflect a range of cancer studies, including malignancies of the skin [14-16], breast [17-19], blood [20], and brain [21]. The full description, accession, and size for each library were recorded [see Additional file 2]. In the case of breast and skin data, libraries from a combination of studies were used. Datasets were filtered to remove tags expressed at a mean less than 100 tags per million.

### Model fitting

The open-source statistical software package R was used to perform all calculations in this paper [22]. R code is included with the explanation for each model. For each of the models, let *Y*_*i *_be the observed tag count in library *i*, *n*_*i *_be the total number of tags in library *i*, and *N *be the total number of libraries. Also, let *x*_i _be the vector of explanatory variables (e.g. normal = 0 and cancer = 1) associated with the library *i*, and *β *be the vector of coefficients.

### Log-linear (Poisson) regression model

The log-linear model assumes that the observed tag counts are distributed as

Y_i _~ Poisson(*μ*_i_)

*μ*_i _= n_i_p_i_

where p_i _is the actual expression in terms of the proportion of all expressed tags.

Here, the unconditional mean and variance are E(Y_i_) = Var(Y_i_) = *μ*_i_. Using the log link function, which linearizes the relationship between the dependent variables and the predictor(s), we obtain the linear equation

log(Y_i_) = log(n_i_) + *x*_i_*β*

p_i _= exp(*x*_i_*β*)

Using iteratively reweighted least-squares (IRLS), the value(s) for the coefficient(s) *β *are estimated. The **stats **library included with R can fit a log-linear model using the following code:

counts <- c(9, 13, 11, 8, 9, 20, 16, 19, 18, 15)

library.sizes <- rep(100000, 10)

# first 5 observations are from sample type 0 (e.g. normal)

# last 5 observations are from sample type 1 (e.g. cancer)

classes <- c(0,0,0,0,0,1,1,1,1,1)

fit <- glm(counts ~ offset(log(library.sizes)) + classes, family=poisson(link="log"))

# get the beta coefficients

beta0 <- fit$coefficients[[1]]

beta1 <- fit$coefficients[[2]]

# get the expression (expressed as a proportion) for each group

prop0 <- exp(beta0)

prop1 <- exp(beta0+beta1)

# calculate significance score for differential expression

# i.e. null hypothesis that beta_1 = 0

t.value <- summary(fit)$coefficients [,"z value"][2]

p.value <- 2 * pt(-abs(t.value), fit$df.residual)

### Overdispersed log-linear regression model

In contrast to the canonical log-linear model, we assume the actual expression is distributed as

*θ*_i _~ Gamma(*μ*_i_, 1/*ϕ*),

*μ*_i _= n_i_p_i_

where, as above, p_i _is the actual expression in terms of the proportion of all expressed tags. Here, the unconditional mean and variance are E(*θ*_i_) = *μ*_i _and Var(*θ*_i_) = *μ*_i_^2^*ϕ*. Since we are now sampling from this latent Gamma distribution, the observed tag counts are conditional on this underlying expression and are distributed as

Y_i _| p_i_,*ϕ *~ Poisson(*θ*_i_)

Now, the unconditional mean and variance are E(Y_i_) = *μ*_i _and Var(Y_i_) = *μ*_i_(1+*μ*_i_*ϕ*).

As above, using the log link function we obtain the linear equation

log(Y_i_) = log(n_i_) + *x*_i_*β*

p_i _= exp(*x*_i_*β*)

Here, a maximum likelihood estimate of the values for the coefficient(s) *β *and the overdispersion parameter (*ϕ*) can be performed. The **MASS **library [23] for R can fit an overdispersed log-linear model using the following code:

library(MASS)

counts <- c(9, 13, 11, 8, 9, 20, 16, 19, 18, 15)

library.sizes <- rep(100000, 10)

# first 5 observations are from sample type 0 (e.g. normal)

# last 5 observations are from sample type 1 (e.g. cancer)

classes <- c(0,0,0,0,0,1,1,1,1,1)

fit <- glm.nb(counts ~ offset(log(library.sizes)) + classes)

# get the beta coefficients

beta0 <- fit$coefficients[[1]]

beta1 <- fit$coefficients[[2]]

# get the dispersion parameter

dispersion <- 1/fit$theta

# get the expression (expressed as a proportion) for each group

prop0 <- exp(beta0)

prop1 <- exp(beta0+beta1)

# calculate significance score for differential expression

# i.e. null hypothesis that beta_1 = 0

t.value <- summary(fit)$coefficients [,"z value"][2]

p.value <- 2 * pt(-abs(t.value), fit$df.residual)

A more complete discussion of this model and its application to SAGE, including significance testing, is described in [6].

### Poisson mixture model

Like the canonical log-linear regression model, we assume the observed tag counts are Poisson distributed. However, the counts are conditional on the choice of a Poisson-distributed component, such that

Y_i _| k ~ Poisson(*μ*_ik_)

*μ*_ik _= n_i_p_ik_

where the component k = 1, 2, ..., K and p_ik _is the actual expression for component k in terms of the proportion of all expressed tags. The posterior probability that an observed tag count belongs to a component k is given by

P(k|Yi,ψ)=πkf(Yi|μik)∑jKf(Yi|μij)
 MathType@MTEF@5@5@+=feaafiart1ev1aaatCvAUfKttLearuWrP9MDH5MBPbIqV92AaeXatLxBI9gBaebbnrfifHhDYfgasaacH8akY=wiFfYdH8Gipec8Eeeu0xXdbba9frFj0=OqFfea0dXdd9vqai=hGuQ8kuc9pgc9s8qqaq=dirpe0xb9q8qiLsFr0=vr0=vr0dc8meaabaqaciaacaGaaeqabaqabeGadaaakeaacqWGqbaucqGGOaakcqWGRbWAcqGG8baFcqWGzbqwdaWgaaWcbaGaemyAaKgabeaakiabcYcaSGGaciab=H8a5jabcMcaPiabg2da9maalaaabaGae8hWda3aaSbaaSqaaiabdUgaRbqabaGccqWGMbGzcqGGOaakcqWGzbqwdaWgaaWcbaGaemyAaKgabeaakiabcYha8jab=X7aTnaaBaaaleaacqWGPbqAcqWGRbWAaeqaaOGaeiykaKcabaWaaabCaeaacqWGMbGzcqGGOaakcqWGzbqwdaWgaaWcbaGaemyAaKgabeaakiabcYha8jab=X7aTnaaBaaaleaacqWGPbqAcqWGQbGAaeqaaOGaeiykaKcaleaacqWGQbGAaeaacqWGlbWsa0GaeyyeIuoaaaaaaa@58EE@

where *ψ *is the parameter vector containing the component means (*θ*_1_,...,*θ*_K_) and mixing coefficients (*π*_1_,...*π*_K-1_). f(.) is the Poisson probability density function. To fit the model, one must estimate the values in *ψ*. This can be done by maximum likelihood estimation (MLE) using the EM algorithm [24]. The **flexmix **library for R uses the EM algorithm to fit a variety of finite mixture models [25]. In the case of SAGE data, the following code can be used:

library(flexmix)

counts <- c(9, 13, 11, 8, 9, 20, 16, 19, 18, 15)

library.sizes <- rep(100000, 10)

# first 5 observations are from sample type 0 (e.g. normal)

# last 5 observations are from sample type 1 (e.g. cancer)

classes <- c(0,0,0,0,0,1,1,1,1,1)

# set fitting control parameters to settings that work

# well for SAGE

custom.FLXcontrol <- list(iter.max=200,

         minprior=0,

         tolerance=1E-6,

         verbose=0,

         classify="hard",

         nrep=1)

custom.FLXcontrol <- as(custom.FLXcontrol, "FLXcontrol")

# specify the maximum number of model components

maxk <- 5

fits <- list()

aic.fits <- rep(NA, maxk)

# increase number of components until AIC decreases

for(k in 1:maxk) {

   # make an initial "good" guess of class membership

   # using k-means – helps avoid falling into a local

   # likelihood maximum

   cm <- rep(1, length(counts))

   if(k > 1) cm <- kmeans((counts+1)/(sizes+2),

         centers=k)$cluster

   fit <- try(flexmix(counts ~ 1,

      k=k,

      model=FLXglm(family="poisson",

            offset=log(sizes)),

      control=custom.FLXcontrol,

      cluster=cm), silent=TRUE)

   if("try-error" %in% class(fit)) break

   # stop if there were less components found then

   # specified

   if(max(cluster(fit)) > k) break

   fits [k]] <- fit

   aic.fits [k] <- AIC(fits [k]])

   if(k == 1) next

   if(aic.fits [k] >= aic.fits [k-1]) break

}

# what number of components minimized AIC?

k.optimal <- which(aic.fits == min(aic.fits, na.rm=TRUE))[1]

fit <- fits [k.optimal]]

# get the theta parameters

thetas <- array(dim=k.optimal)

for(i in 1:k.optimal) thetas [i] <- parameters(fit,component=i)$coef

# get the pi parameters

pis <- attributes(fit)$prior

# what is the confidence score that the fitted components

# differentiate between groups?

confidence <- pmm.confidence(fit, classes, use.scaled=FALSE)

The confidence score, ranging from 50–100%, is explained in the Results section and code for performing the calculation is available [see Additional file 3].

## Authors' contributions

SDZ conceived of, developed, and tested the presented research.

## Supplementary Material

Additional file 1 Derivation of confidence score. A derivation of a confidence score for differential expression based on a Poisson mixture model fit. Click here for file

Additional file 2 Supplementary SAGE library information. An Excel spreadsheet containing accessions, sizes, and descriptions of the libraries included in this study. Click here for file

Additional file 3 Confidence score R function. R source code for a function to calculate the differential expression confidence score based on a Poisson mixture model fit. Click here for file
