# Supplementary material for: Statistical analysis and significance testing of serial analysis of gene expression data using a Poisson mixture model
Source: BMC Bioinformatics. 2007 Aug 2;8:282. doi: 10.1186/1471-2105-8-282 (PMC2147036; doi:10.1186/1471-2105-8-282)
Supplement: Additional file 1 — Derivation of confidence score. A derivation of a confidence score for differential expression based on a Poisson mixture model fit. [file 1471-2105-8-282-S1.pdf]

Derivation of Differential Expression Confidence Score

Scott Zuyderduyn (scottz@bccrc.ca)

May 4, 2007

Given Bayes Theorem:

$$P(A|B) = \frac{P(B|A)P(A)}{P(B)}$$

First, define A as the sample class (e.g. normal, cancer) and B as a mixture component(s). Therefore, re-write Bayes Theorem as:

$$P(\omega|k) = \frac{P(k|\omega)P(\omega)}{P(k)}$$

The probability of sample type ω being part of mixture component k is given as:

$$P(k|\omega) = \sum_j^{\omega} \tau_{jk} \Big/ N_\omega$$

where τ_{jk} is the posterior probability that observation j arose from component k and N_ω is the number of libraries of type ω.

The probability that a sample is of type ω is simply:

$$P(\omega) = N_\omega \Big/ N$$

where N is the total number of libraries.

The probability that a sample arose from mixture component k is given as:

$$P(k) = \sum_j \tau_{jk} \Big/ N$$

Substituting terms, we arrive at:

$$P(\omega|k) = \frac{\sum_j^{\omega} \tau_{jk}}{N_\omega} \frac{N_\omega}{N} \Bigg/ \frac{\sum_j \tau_{jk}}{N}$$

Finally, by canceling like terms, we have the expression:

$$P(\omega|k) = \sum_j^{\omega} \tau_{jk} \Big/ \sum_j \tau_{jk}$$
